# Pyrozone

**Published:** 1897-09

**Authors:** Conrad E. Wettlauffer


ARTICLE V.

### PYROZONE.

CONRAD E. WETTLAUFFER, D. D. S.

Concerning its use for bleaching. The first procedure will be to cleanse the tooth thoroughly from all decay. In case of an incisor, apply the rubber dam to the one to be bleached, and to as many adjacent teeth as may be necessary to obtain space and light. The teeth not to be bleached should be covered with wax or vaseline to prevent the pyrozone from attacking them. If of a soft texture, when cleansed, wash out with 95 per cent. alcohol, placing a stopping of some suitable material up the pulp canal, such as chloro percha, oxichloride of zinc, with fibers of cotton, or whatever you are in the habit of using in your practice.

Of course you are supposed to have previously reduced the canal to an aseptic condition. After drying out thoroughly with alcohol, wash out with aqua ammonia and dry well. Then take a probe, either gold or platinum, twist a pledget of cotton around it, saturate with pyrozone, 25 per cent. solution, and insert in the tooth and canal, squeezing out the pyrozone.

Then apply hot air on both labial and platina surfaces to evaporate quickly, and continue in this way till the tooth is bleached to its natural color. A dense, hard enamel and dentin will occupy twenty, forty or sixty minutes, and in some cases twice as long; a soft dentin I have bleached in ten or fifteen minutes. The ether in which the pyrozone is dissolved, will penetrate the canaliculi and absorb the protoplasmic matter, and some consideration must be given this fact in the subsequent filling of the teeth, to prevent a return of the discoloration.

I would recommend the wiping out of the cavity and canal with either a thin solution of white shellac, gum copal or Canada balsam. The Canada balsam or white shellac will penetrate by capillary attraction quite a distance through the tubuli, and will also make a water-tight coating in any checks of the enamel, which are so often manifest in devitalized teeth.

I have never met with failure in the bleaching of a tooth in the manner here described. Let me cite a case. Mr. A., aged 40 years, came to me with a left central incisor very badly discolored. which had been so for twenty years. I treated the tooth as above for nearly two hours, and succeeded in restoring it to its normal color. The discoloration returned in a marked degree in three or four days. I attributed it to a gold filling in the tooth. I removed the filling, treated the tooth as before for half an hour, and succeeded in restoring it to its natural color, and it has remained in that condition ever since. This to me proved two things, viz : That pyrozone is a good bleaching agent, and also that a gold filling in a tooth to be bleached must be removed.

I have also found pyrozone very useful in the insertion of all kinds of crown and bridge work. We all know that bleeding of the gum gives us great trouble in these operations. I obviate this by applying a 25 per cent. solution of pyrozone to the gums, which acts as a styptic, and in my hands has never resulted unhappily. I also use this remedy in the same manner for the insertion of gold fillings at cervical margins, or two applications rendering the gum perfectly dry for ten or fifteen minutes which is ample time for the insertion of an ordinary filling. In abscess with fistulous opening, I use a three per cent. solution inserted with a hypodermic syringe, forcing the solution through the fistulous opening. Its use is not as painful as that of peroxid of hydrogen. When the alveolus has been perforated by the progress of of supperation, and the soft tissues have not yet sloughed, I advise free lancing over the congested parts, and the insertion of a pledget of cotton, saturated with a 25 per cent. solution, which should be allowed to remain for a few moments.

For a putrescent pulp, I apply a five per cent. solution pyrozone, which at once disinfects, permitting the removal from the canals of disorganized tissue, without the disagreeable odor which is often so offensive to both patient and operator.

I also use a three per cent. pyrozone for the removal of green stain on teeth. Apply it with an atomizer, spraying the teeth. It is efficacious, and pleasant to patient and operator.

For pyorrhea, after removing thoroughly all the salivary and sanguinary deposits from the necks and roots of teeth, $H_2O_2$ will be found the best agent as a chemical cleanser and antiseptic for washing out the pockets, bringing the *debris* to the surface and putting the parts in hygienic condition. I also instruct the patient to use an atomizer, spraying the gums and mouth with a three per cent. solution of pyrozone and listerin, equal parts, three times daily, till the disease is to a great extent conquered.—*Dental Practitioner.*

---

ARTICLE VI.

